# From positive blood culture to microbiological diagnosis in 4 hours by MALDI-TOF mass spectrometry bacterial identification and rapid antibiogram

**DOI:** 10.1186/cc11784

**Published:** 2012-11-14

**Authors:** S Barnini, V Brucculeri, P Morici, A Lupetti, M Campa

**Affiliations:** 1 Azienda Ospedaliero - Universitaria Pisana, Pisa, Italy

## Background

Time is precious for patients and clinicians facing septic events. In recent past years, many efforts have been made to develop rapid and reliable tests, especially by molecular methods, which still have several limits in determining antimicrobial susceptibility. Nowadays, MALDI-TOF mass spectrometry allows almost instantaneous bacterial identification, but antibiotic susceptibility results are usually delayed 24 to 48 hours, compared with the timing of microorganism identification.

## Methods

In this work, two short procedures, one for Gram-negative and one for Gram-positive bacteria, were developed. The two methods were employed to purify bacteria from positive blood cultures (Becton Dickinson) after microscopic examination, and to prepare bacterial cells for identification. Bacteria were then identified by MALDI-TOF mass spectrometry (Bruker Daltonics) with excellent scores, similar to those obtained from agar plate colonies. After identification, short-term broth cultures were inoculated with an aliquot of the recovered bacteria, with or without (growth controls) antibiotics and incubated in an HB&L (Alifax SpA) instrument, which monitored each vial for bacterial growth during the following 3 hours. Absence or reduction in growth in a vial containing a certain antibiotic was interpreted as sensibility, while resistance was assessed by a bacterial growth comparable with the control vial.

## Results

In these pilot experiments 32 Gram-negative (10 *Klebsiella pneumoniae*, six *Escherichia coli*, four *Enterobacter aerogenes*, four *Pseudomonas aeruginosa*, two *Klebsiella oxytoca*, two *Acinetobacter baumannii*, one *Citrobacter freundii*, one *Proteus mirabilis*, one *Serratia marcescens*, one *Acinetobacter lwoffii*) and 46 Gram-positive (28 *Staphylococcus epidermidis*, eight *Staphylococcus hominis*, three *Staphylococcus capitis*, two *Staphylococcus haemolyticus*, two *Staphylococcus aureus*, one *Staphylococcus auricularis*, one *Enterococcus faecalis*, one *Enterococcus faecium*) bacterial isolates were correctly identified and 274 rapid susceptibility tests were performed. Among these, 259 were compared with results from Vitek 2 (bioMérieux), obtained 48 hours later from agar-plated cultures of routine analysis, with an overall agreement of 93% (1.5% very major errors, 4% major errors, 1% minor errors; discrepancies resolved by E-test). Antibiotics tested were chosen among cefotaxime, ceftazidime, gentamicin, levofloxacin, meropenem, amikacin and colistin for Gram-negative bacteria and cefoxitin, linezolid, teicoplanin and ampicillin for Gram-positive bacteria. All but cefoxitin gave results in 3 hours.

## Conclusion

These results show that rapid and reliable microbiological answers can be given in a few hours, thus allowing clinicians to start a proper and lifesaving antibiotic therapy.

